# Radiation-induced resistance oscillations in 2D electron systems with strong Rashba coupling

**DOI:** 10.1038/s41598-017-14125-1

**Published:** 2017-10-19

**Authors:** Jesús Iñarrea

**Affiliations:** 1 Escuela Politécnica Superior, Universidad Carlos III, Leganes, Madrid, Spain; 2 Unidad Asociada al Instituto de Ciencia de Materiales, CSIC Cantoblanco, Madrid, 28049 Spain

## Abstract

We present a theoretical study on the effect of radiation on the mangetoresistance of two-dimensional electron systems with strong Rashba spint-orbit coupling. We want to study the interplay between two well-known effects in these electron systems: the radiation-induced resistance oscillations and the typical beating pattern of systems with intense Rashba interaction. We analytically derive an exact solution for the electron wave function corresponding to a total Hamiltonian with Rashba and radiation terms. We consider a perturbation treatment for elastic scattering due to charged impurities to finally obtain the magnetoresistance of the system. Without radiation we recover a beating pattern in the amplitude of the Shubnikov de Hass oscillations: a set of nodes and antinodes in the magnetoresistance. In the presence of radiation this beating pattern is strongly modified following the profile of radiation-induced magnetoresistance oscillations. We study their dependence on intensity and frequency of radiation, including the teraherzt regime. The obtained results could be of interest for magnetotransport of nonideal Dirac fermions in 3D topological insulators subjected to radiation.

## Introduction

Radiation-induced resistance oscillations (RIRO) and zero resistance states (ZRS)^1,2^ are remarkable phenomena in condensed matter physics that reveal a novel scenario in radiation-matter coupling. Those effects rise up when a high-mobility, typically above 10^6^ *cm* ^2^/*Vs*, two dimensional electron system under a moderate magnetic field (*B*) is illuminated with microwave (MW) or terahertz (TH) radiation. The mangetoresistance (*R*_*xx*_) of such systems (2DES) shows oscillations with peaks and valleys at a certain radiation power. When increasing power, the *R*_*xx*_ oscillations increase in turn and at high enough intensity the valleys turn into ZRS. The radiation-induced resistance oscillations show characteristic traits such as periodicity in the inverse of *B* ^1,2^, a 1/4 cycle shift in the oscillations minima^3^, sensitivity to temperature^4,5^ and radiation power^6^. For the latter case, a sublinear law is obtained for the dependence of RIRO on the radiation power, *R*_*xx*_ ∝ *P* ^*α*^, where *P* is the radiation power and, interestingly, the exponent is around 0.5. This clearly indicates a squared root dependence^7–16^.

A great number of experiments and theoretical models have been presented to date to try to explain such striking effects. From a theoretical standpoint, we can cite for instance the displacement model^17^ based on radiation-assisted inter Landau level scattering, the inelastic model based on the effect of radiation on the nonequilibrium electron distribution function^18^. Being these two models the most cited to date, other models are also very successful explaining the basic features of RIRO, such as the one by Lei *et al*.^19^, or the radiation-driven electron orbit model^20–24^. We have to admit that to date there is no universally accepted theoretical approach among the people devoted to this field. The current or future theoretical models dealing with RIRO or ZRS have to confront with the available experimental results to prove how good and accurate they are. Another approach to prove theories is to check out if they are able to predict results on novel scenarios where there are no experiments carried out yet. For instance RIRO and ZRS obtained on different semiconductor platforms other than GaAs, the most extensively platform used in this kind of experiments. The main reason for using GaAs is that this platform offers the highest mobility^25^ to date, (~3.0×10^7^ *cm* ^2^/*Vs*), among different semiconductor heterostructures.

In this article we present a theoretical study on the effect of radiation on the magnetotransport in samples with strong Rashba^26–28^ spin-orbit interaction (RSOI), such as InAs. The interest by heterostructures with InAs is increasing very fast in the last years, on the one hand for their technological impact being part of new electronic devices. On the other hand from basic research standpoint in fields such as spintronics transistors and the realisation of Majorana fermions^29^. Usually, the electron mobility in InAs has been always below 1.0 × 10^6^ *cm* ^2^/*Vs* and RIRO can hardly be seen. Yet, there have been published very recently experimental results demonstrating that improving MBE growth techniques in quantum wells of InAs electron mobilities can be dramatically increased^30^. They claimed a mobility close to 3.0 × 10^6^ *cm* ^2^/*Vs*. Therefore, samples of InAs, with strong RSOI, can now become reasonable candidates to observe RIRO. Then, we could study the interplay of Rashba interaction and radiation in these kind of systems. We could also predict that with samples with even higher mobilities and at high enough radiation intensity, 2DES systems with RSOI can give rise to ZRS.

Thus, we start off based on the previous theory of *the radiation-driven electron orbit* ^20–24^. This theory stems from the displacement model^17^ and shares with it the interplay between charged impurity scattering and radiation to be at the heart of RIRO. As a further evolution of the displacement model, our theory proceeds in an alternative approach starting from the exact solution of the time-dependent Schrodinger equation for an electron under magnetic field and radiation. The obtained exact wave function represents a Landau state where the guiding center is harmonically driven back and forth by radiation at the same frequency. Interestingly, the Landau states guiding center follows a classical trajectory given by the solution of the driven classical oscillator. According to this theory, the interaction of the driven Landau states with charged impurities ends up giving rise to shorter and longer average advanced distances by the scattered electrons. These distances are reflected on irradiated *R*_*xx*_ as valleys and peaks respectively.

We have added to the same total Hamiltonian of the radiation-driven electron orbit theory the Rashba interaction, solving exactly the corresponding time-dependent Schrodinger equation. Then, applying a Boltzmann transport model we are able to obtain an expression for *R*_*xx*_ with radiation and RSOI. In the simulations we obtain, first without radiation, the well-known beating pattern with the system of nodes and antinodes of the Rashba magnetoresistance^31–43^. Then, we switched on light obtaining *R*_*xx*_ that shows a strong deformation of the previous beating pattern where the nodes and antinodes follow the peaks and valleys of RIRO. We study the dependence on power and frequency including the terahertz regime. 2DES with RSOI share similar Hamiltonian with 3D topological insulators, then we consider that the results that we present in this article could be of application in the field of 3D topological insulators under the influence of radiation.

## Theoretical Model

We consider a 2DES in the *x*–*y* plane with strong Rashba coupling subjected to a static and perpendicular *B* and a DC electric field parallel to the *x* direction. Using Landau gauge for the potential vector, **A** = (0, *Bx*, 0), the hamiltonian of such a system, *H*_0_ reads: *H*_0_ = (*H*_*B*_ + *H*_*SO*_) where the different components of *H*_0_ are:

1 $$\begin{array}{rcl}{H}_{B} & = & [\frac{{p}_{x}^{2}}{2{m}^{\ast }}+\frac{1}{2}{m}_{\ast }{w}_{c}^{2}{(x-{X}_{0})}^{2}]\,{\sigma }_{0}+\frac{1}{2}g{\mu }_{B}{\sigma }_{z}B\\  &  & +[-e{E}_{dc}{X}_{0}+\frac{1}{2}{m}^{\ast }\frac{{E}_{dc}^{2}}{{B}^{2}}]\,{\sigma }_{0}\end{array}$$

2 $${H}_{SO}=-\frac{\alpha }{\hslash }\,[{\sigma }_{y}{p}_{x}-{\sigma }_{x}eB(x-{X}_{0})]$$

*X*_0_ is the center of the orbit for the electron spiral motion: ${X}_{0}=-(\frac{\hslash {k}_{y}}{eB}-\frac{e{E}_{dc}}{{m}^{\ast }{w}_{c}^{2}})$, *E*_*dc*_ is the DC electric field parallel to the *x* direction, *σ*_0_ stands for the unit matrix, $\overrightarrow{\sigma }=({\sigma }_{x},{\sigma }_{y},{\sigma }_{z})$ are the Pauli spin matrices, *g* the Zeeman factor, *μ*_*B*_ the Bhor magneton, *α* the Rashba spin-orbit coupling parameter and *w*_*c*_ the cyclotron frequency. The Schrodinger equation corresponding to the Hamiltonian *H*_0_ can be exactly solved and the resulting states are labeled by the quantum number *N*. For *N* = 0 there is only one level of energy given by *E*_0_ = (*ħw*_*c*_ − *gμ*_*B*_ *B*)/2. For *N* ≥ 1 we obtain two branches of levels labelled by + and − and with energies:

3 $${E}_{N\pm }=\hslash {w}_{c}N\pm \frac{1}{2}\sqrt{{(\hslash {w}_{c}-g{\mu }_{B}B)}^{2}+\frac{8{\alpha }^{2}}{{R}^{2}}N}$$

where *R* is the magnetic length, $R=\sqrt{\frac{\hslash }{eB}}$. The corresponding wave function for the + branch is,

4 $${\psi }_{N+}=\frac{1}{\sqrt{{L}_{y}}}{e}^{i{k}_{y}y}\,(\begin{array}{c}\cos \,\frac{\theta }{2}{\varphi }_{N-1}\\ \sin \,\frac{\theta }{2}{\varphi }_{N}\end{array})$$

and for the - branch,

5 $${\psi }_{N-}=\frac{1}{\sqrt{{L}_{y}}}{e}^{i{k}_{y}y}\,(\begin{array}{c}-\sin \,\frac{\theta }{2}{\varphi }_{N-1}\\ \cos \,\frac{\theta }{2}{\varphi }_{N}\end{array})$$

where *θ* is given by $\theta =\arctan \,[\tfrac{2\tfrac{\sqrt{2}}{R}\alpha \sqrt{N}}{g{\mu }_{B}B-\hslash {w}_{c}}]$ and *ϕ* is the normalized quantum harmonic oscillator wave function, i.e., Landau state, *N* being the corresponding Landau level index. According to these results the Rashba spin-orbit interaction mixes spin-down and spin-up states of adjacent Landau levels to give rise to two new energy branches of eigenstates of the Hamiltonian *H*_0_.

To analyze magnetotransport in 2DES with RSOI we calculate the longitudinal conductivity *σ*_*xx*_ following the Boltzmann transport theory^44–46^, where *σ*_*xx*_ is given by:

6 $${\sigma }_{xx}={e}^{2}\,{\int }_{0}^{\infty }\,dE{\rho }_{i}(E)\,{[{\rm{\Delta }}X\mathrm{(0)]}}^{2}{W}_{I}\,(-\frac{df(E)}{dE})$$

being *E* the energy, *ρ*_*i*_(*E*) the density of initial states and *f*(*E*) the electron distribution function. Δ*X*(0) is the shift of the guiding center coordinate for the eigenstates involved in the scattering event, or in other words, the averaged advanced distance by the scattered electron when jumping between initial and final LS,

7 $${\rm{\Delta }}X\mathrm{(0)}=[{X}_{2}\mathrm{(0)}-{X}_{1}\mathrm{(0)]}\simeq 2{R}_{c}$$

*X*_2_(0) and *X*_1_(0) being the guiding center coordinates for final and initial states respectively and *R*_*c*_ the cyclotron radius. *W*_*I*_ is the remote charged impurity scattering rate because we consider that at very low temperatures (*T*) this is the most likely source of scattering for electrons in high mobility 2DES. According to the Fermi’s Golden Rule *W*_*I*_ is given by

8 $${W}_{I}=\frac{2\pi }{\hslash }{N}_{I}{|\langle {\psi }_{f\pm }|{V}_{s}|{\psi }_{i\pm }\rangle |}^{2}\delta ({E}_{f}-{E}_{i})$$

where *N*_*I*_ is the impurity density and *E*_*i*_ and *E*_*f*_ are the energies of the initial and final states respectively. *V*_*s*_ is the scattering potential for charged impurities^45^. The matrix element inside *W*_*I*_ can be expressed as^44–46^:

9 $${|\langle {\psi }_{f\pm }|{V}_{s}|{\psi }_{i\pm }\rangle |}^{2}=\sum _{q}\,{|{V}_{q}|}^{2}{|{I}_{if}|}^{2}{\delta }_{{k}_{y}^{^{\prime} },{k}_{y}+{q}_{y}}$$

where ${V}_{q}=\tfrac{{e}^{2}}{\epsilon (q+{q}_{s})}$, $\epsilon$ the dielectric constant and *q*_*s*_ is the Thomas-Fermi screening constant^45^. The integral *I*_*if*_ is given by:

10 $${I}_{if}=\frac{1}{2}\,{\int }_{-\infty }^{\infty }\,[\pm {{\rm{\Phi }}}_{f-1},{{\rm{\Phi }}}_{f}]\,(\begin{array}{cc}{e}^{i{q}_{x}x} & 0\\ 0 & {e}^{i{q}_{x}x}\end{array})\,[\begin{array}{c}\pm {{\rm{\Phi }}}_{i-1}\\ {{\rm{\Phi }}}_{i}\end{array}]\,dx$$

where we have considered that at low or moderate *B* (used in experiments of magnetoresistance oscillations) $[\tfrac{2\tfrac{\sqrt{2}}{R}\alpha \sqrt{N}}{g{\mu }_{B}B-\hslash {w}_{c}}]\to \infty$ and then $\theta \simeq \tfrac{\pi }{2}$

To calculate the density of states *ρ*_*i*_(*E*) of a 2DES with perpendicular *B* and RSOI we proceed starting off with the expression of the energy of the states, eq. (3) that can be rewritten in a more compact way:

11 $${E}_{N\pm }=\hslash {w}_{c}\,[N\pm \sqrt{\tfrac{1}{4}+\hslash 2N{\tilde{\alpha }}^{2}}]$$

where ${\tilde{\alpha }}^{2}={\alpha }^{2}\frac{{m}^{\ast }}{{\hslash }^{4}{w}_{c}}$. To obtain the new expression for *E*_*N*±_ we have neglected the Zeeman term considering that at the magnetic fields used in experiments and in simulations it is much smaller than the Rashba term^35^. Expressing the density of states in terms of Dirac *δ*-function we can write:

12 $$\begin{array}{rcl}{\rho }_{i}(E) & = & \frac{eB}{h}\delta (E-{E}_{0})\\  &  & +\frac{eB}{h}\,\sum _{N=1}^{\infty }\,[\delta (E-{E}_{N+})+\delta (E-{E}_{N-})]\end{array}$$

where ${E}_{0}=\tfrac{\hslash {w}_{c}}{2}$. To do the sum in the expression of *ρ*_*i*_ we use the Poisson sum rules,

13 $$\sum _{n=1}^{\infty }\,f(n)=-\frac{1}{2}f\mathrm{(0)}+{\int }_{0}^{\infty }\,f(x)dx+2\,\sum _{s=1}^{\infty }\,{\int }_{0}^{\infty }\,\cos \,\mathrm{(2}\pi sx)\,f(x)dx$$

and after some lengthy algebra we get to an expression that includes the state broadening and reads^47,48^:

14 $$\begin{array}{rcl}{\rho }_{i}(E) & = & \frac{{m}^{\ast }}{\pi {\hslash }^{2}}\{1+\sum _{\pm }\,(1\pm \frac{\hslash {\tilde{\alpha }}^{2}}{\sqrt{\frac{1}{4}+\frac{2E{\tilde{\alpha }}^{2}}{{w}_{c}}+{\hslash }^{2}{\tilde{\alpha }}^{4}}})\\  &  & \times \sum _{s=1}^{\infty }\,{e}^{\frac{-s\pi {\rm{\Gamma }}}{\hslash {w}_{c}}}\,\cos \,[2\pi s\,(\frac{E}{\hslash {w}_{c}}+\hslash {\tilde{\alpha }}^{2}\pm \sqrt{\frac{1}{4}+\frac{2E{\tilde{\alpha }}^{2}}{{w}_{c}}+{\hslash }^{2}{\tilde{\alpha }}^{4}})]\}\end{array}$$

Γ, being the sates width. This equation is essential in the present article because it reveals the presence of two cosine terms that could interfere. On the other hand, it also important to highlight that it is obtained from an expression for the states energy that depends at the same time on the “Landau” level index, both linearly and through a square root. With this expression of the states density we recover the previous one obtained by Ch. Amann^47^ including the states broadening. This last condition makes the expression much more useful to be used in theories explaining experimental results on 2DES with RSOI. Considering that only electrons around the Fermi level participate in the magnetotransport and the usual electron density used in these experiments^30^, it turns out that the *E* term is much bigger than the Rashba term. Therefore, we can rewrite the expression of the density of states as:

15 $$\begin{array}{rcl}{\rho }_{i}(E) & = & \frac{{m}^{\ast }}{\pi {\hslash }^{2}}\,\{1+\sum _{s=1}^{\infty }\,{e}^{\frac{-s\pi {\rm{\Gamma }}}{\hslash {w}_{c}}}\,[\cos \,2\pi s\,(\frac{E}{\hslash {w}_{c}}+\sqrt{\frac{1}{4}+\frac{2E{\tilde{\alpha }}^{2}}{{w}_{c}}})\\  &  & +\,\cos \,2\pi s\,(\frac{E}{\hslash {w}_{c}}-\sqrt{\frac{1}{4}+\frac{2E{\tilde{\alpha }}^{2}}{{w}_{c}}})]\}\end{array}$$

Finally and after some algebra we can write an expression for *σ*_*xx*_,

16 $$\begin{array}{rcl}{\sigma }_{xx} & = & \frac{{e}^{2}{m}^{\ast }}{\pi {\hslash }^{2}}{({\rm{\Delta }}{X}_{0})}^{2}{W}_{I}\,\{1+\sum _{s=1}^{\infty }\,{e}^{\frac{-s\pi {\rm{\Gamma }}}{\hslash {w}_{c}}}\,\frac{{X}_{S}}{\sinh \,{X}_{S}}\,[\cos \,2\pi s\,(\frac{{E}_{F}}{\hslash {w}_{c}}+\sqrt{\frac{1}{4}+\frac{2{E}_{F}{\tilde{\alpha }}^{2}}{{w}_{c}}})\\  &  & +\,\cos \,2\pi s\,(\frac{{E}_{F}}{\hslash {w}_{c}}-\sqrt{\frac{1}{4}+\frac{2{E}_{F}{\tilde{\alpha }}^{2}}{{w}_{c}}})]\}\end{array}$$

where *E*_*F*_ stands for the Fermi energy and ${X}_{S}=\frac{2{\pi }^{2}{k}_{B}T}{\hslash {w}_{c}}$, *k*_*B*_ being the Boltzmann constant. To obtain *R*_*xx*_ we use the relation ${R}_{xx}=\frac{{\sigma }_{xx}}{{\sigma }_{xx}^{2}+{\sigma }_{xy}^{2}}\simeq \frac{{\sigma }_{xx}}{{\sigma }_{xy}^{2}}$, where ${\sigma }_{xy}\simeq \frac{{n}_{i}e}{B}$ and ${\sigma }_{xx}\ll {\sigma }_{xy}$, *n*_*i*_ being the 2D electron density. The sum of cosine terms in the expression of *σ*_*xx*_ will give rise to an interference effect that will become apparent as a beating pattern. Thus, the physical origin of the beating pattern, that has been experimentally observed, can be trace back to the slightly different energies of the two eigenstates branches.

The Hamiltonian *H*_0_ is the same as the one of the surface states of nonideal Dirac fermions in 3D topological insulators. The only difference is that for the latter the quadratic term is small compared to linear term that it is the dominant when it comes to topological insulators. In real samples the surface states of 3D topological insulators are no longer described by massles Dirac fermions. Experiments demonstrate important band bending and broken electron-hole symmetry with respect to the Dirac point in the band structure of real 3D topological insulators^49,50^. Therefore the results presented above, especially the ones concerning density of states and states energy, could be of interest in the study of magnetotransport in real 3D topological insulators.

If now we switch on radiation, first of all we have to add to the Hamiltonian *H*_0_ a radiation term *H*_*R*_ and then: *H*_0_ = (*H*_*B*_ + *H*_*SO*_ + *H*_*R*_), where

17 $${H}_{R}=-(x-{X}_{0}){\varepsilon }_{0}\,\cos \,wt-{X}_{0}{\varepsilon }_{0}\,\cos \,wt$$

*ε*_0_ being the radiation electric field and *w* the corresponding radiation frequency. *H*_0_ can again be solved exactly^20,21,51,52^, and the solution for the electronic wave function is made up, as above, of two states branches. The wave function for the + branch is,

18 $${{\rm{\Psi }}}_{N+}=\frac{1}{\sqrt{{L}_{y}}}{e}^{i{k}_{y}y}\,(\begin{array}{c}\cos \,\frac{\theta }{2}{{\rm{\Psi }}}_{N-1}(x,t)\\ \sin \,\frac{\theta }{2}{{\rm{\Psi }}}_{N}(x,t)\end{array})$$

and for the - branch,

19 $${{\rm{\Psi }}}_{N-}=\frac{1}{\sqrt{{L}_{y}}}{e}^{i{k}_{y}y}\,(\begin{array}{c}-\sin \,\frac{\theta }{2}{{\rm{\Psi }}}_{N-1}(x,t)\\ \cos \,\frac{\theta }{2}{{\rm{\Psi }}}_{N}(x,t)\end{array})$$

where,

20 $$\begin{array}{rcl}{{\rm{\Psi }}}_{N}(x,t) & = & {{\rm{\Phi }}}_{N}(x-X\mathrm{(0)}-{x}_{cl}(t),t)\\  &  & \times {e}^{[i\tfrac{{m}^{\ast }}{\hslash }\tfrac{d{x}_{cl}(t)}{dt}[x-{x}_{cl}(t)]+\tfrac{i}{\hslash }{\int }_{0}^{t}Ldt^{\prime} ]}\end{array}$$

as above, Φ_*n*_ is the solution for the Schrödinger equation of the unforced quantum harmonic oscillator where *x*_*cl*_(*t*) is the classical solution of a forced harmonic oscillator^20,51,52^,

21 $$\begin{array}{rcl}{x}_{cl}(t) & = & \frac{e{\varepsilon }_{o}}{{m}^{\ast }\sqrt{{({w}_{c}^{2}-{w}^{2})}^{2}+{\gamma }^{4}}}\,\cos \,(wt-\beta )\\  & = & A\,\cos \,(wt-\beta )\end{array}$$

where *e* is the magnitude of the electron charge and *γ* is a phenomenologically introduced damping factor for the electronic interaction with acoustic phonons. *β* is the phase difference between the radiation-driven guiding center and the driving radiation itself. *L* with RSOI is now given by,

22 $$L=\frac{1}{2}{m}^{\ast }{\dot{x}}_{cl}^{2}-\frac{1}{2}{m}^{\ast }{w}_{c}^{2}{x}_{cl}^{2}-\frac{\alpha }{\hslash }\,[{\sigma }_{y}{m}^{\ast }{\dot{x}}_{cl}-{\sigma }_{x}eB{x}_{cl}]$$

Apart from phase factors, the wave function for *H*_0_ now is the same as the standard harmonic oscillator where the center is displaced by *x*_*cl*_(*t*). In the presence of radiation, the electronic orbit center coordinates change and are given according to our model by *X*(*t*) = *X*(0) + *x*_*cl*_(*t*). This means that due to the radiation field all the electronic orbit centers in the sample harmonically oscillate at the radiation frequency in the *x* direction through *x*_*cl*_. Applying initial conditions, at *t* = 0, *X*(*t*) = *X*(0) and then *β* = *π*/2. As a result the expression for the time dependent guiding center is now:

23 $$X(t)=X\mathrm{(0)}+A\,\sin \,wt$$

**Figure 1:**
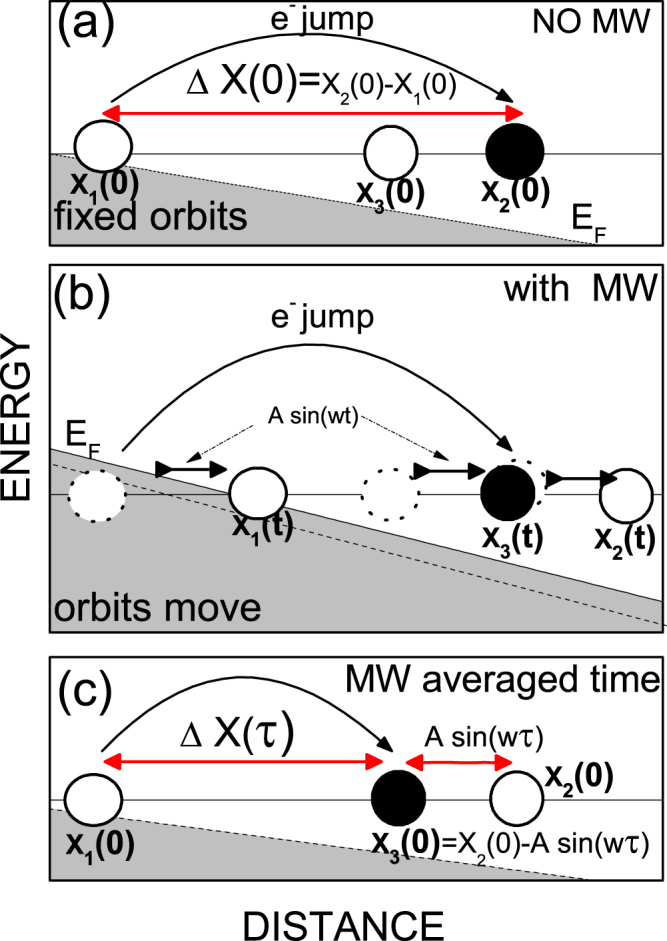
Schematic diagrams for elastic scattering (charged impurities) between Landau States without radiation (Fig. 1a), with radiation where all Landau States move at radiation frequency (Fig. 1b) and with radiation but in the steady state after time average. In this description the final average advanced distance, under radiation and after time average, turns out to be smaller than in the dark. Thus, we obtain a valley in the radiation-induced oscillations. Similar situation but in reverse can be depicted for a peak.

In the presence of charged impurities scattering and radiation the average advanced distance by electrons is going to be different than in the dark, Δ*X*(0) = [*X*_2_(0) − *X*_1_(0)] (see Fig. 1a). Now the positions of the Landau states guiding centers are time-dependent according to the last expression (Eq. 23). If the scattering event begins at a certain time *t*, the initial LS is given by, *X*_1_(*t*) = *X*_1_(0) + *A* sin *wt*. After a time *τ*, that we call *flight time*, the electron “lands” in a final LS that is no longer *X*_2_ as in the dark scenario; due to the swinging nature of irradiated LS, its former position is taken now by a new LS that we can call *X*_3_ (see Fig. 1b). Thus, the new final LS under irradiation is written as, *X*_3_(*t* + *τ*) = *X*_3_(0) + *A* sin *w*(*t* + *τ*), and the scattering-induced advanced distance by the electron reads now,

24 $$\begin{array}{rcl}{\rm{\Delta }}X(t) & = & {X}_{3}(t+\tau )-{X}_{1}(t)\\  & = & {X}_{3}\mathrm{(0)}+A\,\sin \,w(t+\tau )-{X}_{1}\mathrm{(0)}-A\,\sin \,wt\end{array}$$

In order to obtain the steady-state regime for the advanced distance we time-average over a period of the radiation field,

25 $$\begin{array}{rcl}\langle {\rm{\Delta }}X(t)\rangle  & = & \langle {X}_{3}(t+\tau )-{X}_{1}(t)\rangle \\  & = & {X}_{3}\mathrm{(0)}+\langle A\,\sin \,w(t+\tau )\rangle \\  &  & -{X}_{1}\mathrm{(0)}-\langle A\,\sin \,wt\rangle \\  & = & {X}_{3}\mathrm{(0)}-{X}_{1}\mathrm{(0)}\end{array}$$

where obviously we have taken into account that 〈*A* sin *w*(*t* + *τ*)〉 = 0 and 〈*A* sin *wt*〉 = 0. Here, the angular brackets describe time-average over a period of the time-dependent field.

Next, we have to relate 〈Δ*X*(*t*)〉 with the advanced distance in the dark Δ*X*(0), i.e., we have to express *X*_3_(0) in terms of *X*_2_(0). Since during the time *τ* all LS have been displaced in phase the same distance, *A* sin *wτ*, the condition to be fulfilled by those guiding centers is that *X*_3_(0) and *X*_2_(0) have to be separated by *A* sin *wτ*. Then *X*_3_(0) = *X*_2_(0) − *A* sin *wτ* (see Fig. 1c). Substituting this result in the above expression we obtain,

26 $$\begin{array}{rcl}\langle {\rm{\Delta }}X(t)\rangle  & \equiv  & {\rm{\Delta }}X(\tau )=({X}_{2}\mathrm{(0)}-A\,\sin \,w\tau )-{X}_{1}\mathrm{(0)}\\ \,\,\,{\rm{\Delta }}X(\tau ) & = & {\rm{\Delta }}X\mathrm{(0)}-A\,\sin \,w\tau \end{array}$$

According to our model, this expression is responsible of RIRO including the maxima and minima positions. In Fig. 1 we exhibit a schematic description of the scattering process between LS in the dark (Fig. 1a), in the presence of radiation (Fig. 1b), and in the steady state scenario (Fig. 1c). The specific case of Fig. 1 corresponds to a valley in RIRO since the final average advanced distance in the scattering turns out to be smaller than in the dark. Similar description can be given for a peak but now the advanced distance is larger than in the dark.

For the flight time *τ* it was previously proposed, in a quantum mechanical and semiclassical approach^53,54^, that during the scattering jump from one driven orbit to another (in a time *τ*) electrons in their orbits would complete, on average, one full loop, which implies that $\tau ={T}_{c}=\frac{2\pi }{{w}_{c}}$. On the other hand, another way to obtain the expression of *τ* is to compare the condition fulfilled by the minima positions obtained from the theoretical expression (Eq. 26), to the one obtained in experiments^1^. These minima positions represent one of the main traits describing RIRO and were first found by Mani *et al*.^1^ being given by:

27 $$\frac{w}{{w}_{c}}=\frac{5}{4},\frac{9}{4},\frac{13}{4}\mathrm{.}\ldots =(\frac{1}{4}+n)$$

where *n* = 1, 2, 3.…. According to theory, i.e., expression (26), the minima positions are obtained when,

28 $$w\tau =\frac{\pi }{2}+2\pi n\Rightarrow w=\frac{2\pi }{\tau }\,(\frac{1}{4}+n)$$

Then, comparing both expressions we readily obtain that the flight time is,

29 $$\tau =\frac{2\pi }{{w}_{c}}$$

In other words, *τ* equals the cyclotron period *T*_*c*_.

Finally, the advanced distance due to scattering in the presence of radiation reads,

30 $${\rm{\Delta }}X(\tau )={\rm{\Delta }}X\mathrm{(0)}-A\,\sin \,(2\pi \frac{w}{{w}_{c}})$$

Applying these last results to a Boltzmann transport model, similarly as the first part of this section, we can get to an expression for the longitudinal conductivity of the magnetotransport of a high mobility 2DES with strong RSOI in the presence of radiation. In this expression three harmonic terms turn up, two cosine terms depending on the Fermi energy and *α*, that interfere to give rise to the beating pattern profile in the magnetoresistance. And one sine term depending on radiation parameters, frequency and power. We expect the latter to interfere on the beating pattern profile.

31 $$\begin{array}{lll}{\sigma }_{xx} & \propto  & {[{\rm{\Delta }}{X}^{0}-A\sin (2\pi \frac{w}{{w}_{c}})]}^{2}\,\{1+{e}^{\frac{-\pi {\rm{\Gamma }}}{\hslash {w}_{c}}}\,\frac{{X}_{S}}{\sinh \,{X}_{S}}\,[\cos \,2\pi \,(\frac{{E}_{F}}{\hslash {w}_{c}}\\  &  & +\sqrt{\frac{1}{4}+\frac{2{E}_{F}{\tilde{\alpha }}^{2}}{{w}_{c}}})+\,\cos \,2\pi \,(\frac{{E}_{F}}{\hslash {w}_{c}}-\sqrt{\frac{1}{4}+\frac{2{E}_{F}{\tilde{\alpha }}^{2}}{{w}_{c}}})]\}\end{array}$$

where we have considered only the term *s* = 1, the most important, in the sum. We consider that the above results can be of application and can predict the behavior of magnetotransport in 3D topological insulators subjected to radiation; once these systems reach enough mobility to make patent the rise of RIRO. This could happen at the same time that, without radiation, the *R*_*xx*_ beating patter begins to be visible in magnetotransport experiments in 3D topological insulators.

## Results and Discussion

All calculated results presented in this article are based on the next list of parameteres regarding experiments in InAs quantum wells^30,33–37^: Rashba parameter *α* = 0.6 × 10^−11^ *eV* · *m*, electron density *n*_*i*_ = 2.0 × 10^16^ *m* ^−2^, electron effective mass m* = 0.045 *m*_*e*_ where *m*_*e*_ is the electron rest mass and temperature, *T* = 1 *K*.

**Figure 2:**
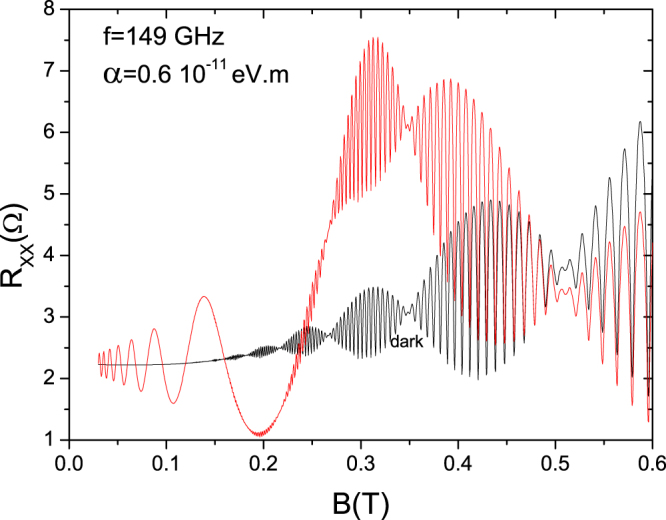
Calculated magnetoresistance, *R*_*xx*_, vs magnetic field, *B*, for dark and radiation of frequency *f* = 149 GHz, in a high-mobility 2DES with strong RSOI of Rashba parameter *α* = 0.6 × 10^−11^ *eV* · *m*. For the dark curve we obtain, as expected, a beating pattern profile made up of a system of nodes and antinodes. The radiation curve exhibits similar beating pattern but modulated by the rise of the system of peaks and valleys of RIRO. (T = 1 K).

In Fig. (2) we present calculated *R*_*xx*_ vs *B* for dark and irradiated scenarios in a high-mobility 2DES with strong RSOI. For the latter, the radiation frequency *f* = 149 GHz. For the dark curve we obtain a very clear beating pattern profile made up of a system of nodes and antinodes. The radiation curve exhibits a similar beating pattern but this time dramatically deformed and modulated by the rise of the system of peaks and valleys of RIRO. In the new beating pattern the node *B*-positions are not affected by the presence of radiation but yet the different antinodes are, according to their *B*-position^55–57^. This peculiar profile in *R*_*xx*_ shows up as result of the interference effect between the sine and cosine terms that is reflected in equation (31).

**Figure 3:**
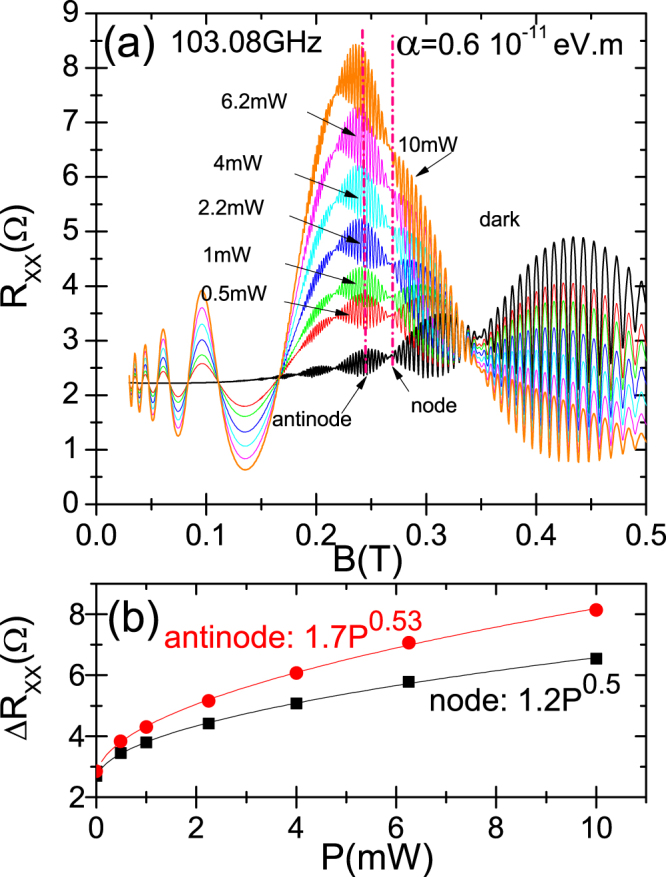
Dependence on radiation power *P* of the calculated magnetoresistivity under light in 2DES with Rashba coupling. In panel (a) we exhibit *R*_*xx*_ as a function of *B*, for different radiation intensities starting from dark and for the same frequency *f* = 103.08 GHz. In panel (b) we exhibit Δ*R*_*xx*_ = *R*_*xx*_ − *R*_*xx*_(*dark*) versus *P* for *B* corresponding to dashed vertical lines on panel (a). One line corresponds to the *B*-position of a node and the other of an antinode. For both, node and antinode, we obtain a square root (sublinear) dependence showing the corresponding fits. (T = 1 K).

In Fig. (3) we present the dependence of calculated *R*_*xx*_ on *P* for 2DES with important Rashba coupling under radiation. In panel (a) we exhibit calculated *R*_*xx*_ vs *B* for a radiation frequency *f* = 103.08 GHz, different radiation intensities from dark to 10 mW: 0.5, 1, 2.2, 4, 6.2 and 10 mW and *T* = 1 K. We easily observe, as expected, that RIRO increase their amplitudes as *P* increases from dark. At the same time the deformation of antinodes gets stronger too, keeping constant the *B*-position of the nodes. In panel (b) we exhibit, again for *f* = 103.08 GHz, Δ*R*_*xx*_ = *R*_*xx*_ − *R*_*xx*_(*dark*) versus *P* for *B* corresponding to dashed vertical lines on panel (a). One line corresponds to the *B*-position of a node and the other of an antinode. We want to check out if the presence of Rashba coupling affects the previously obtained sublinear power law for the dependence of RIRO on *P*. In this way we obtain for both, according to the calculated fits, an approximately square root dependence on *P*, concluding that Rashba coupling does not affect the sublinear law. We can theoretically explain these results according to our model. In the expression of *σ*_*xx*_ and then in *R*_*xx*_, *P* only shows up in the numerator of the amplitude *A* as $\sqrt{P}\propto {\varepsilon }_{0}$, but not in the phase of the sine function. Thus, on the one hand, *P* does not affect the phase of RIRO that remains constant as *P* changes, and on the other hand ${R}_{xx}\propto \sqrt{P}$, giving rise to the sublinear (square root) power law for the dependence of *R*_*xx*_ on *P*. Finally, in the phase of cosine terms there is no radiation parameters concluding that radiation will not affect the *B*-positions of nodes and antinodes.

**Figure 4:**
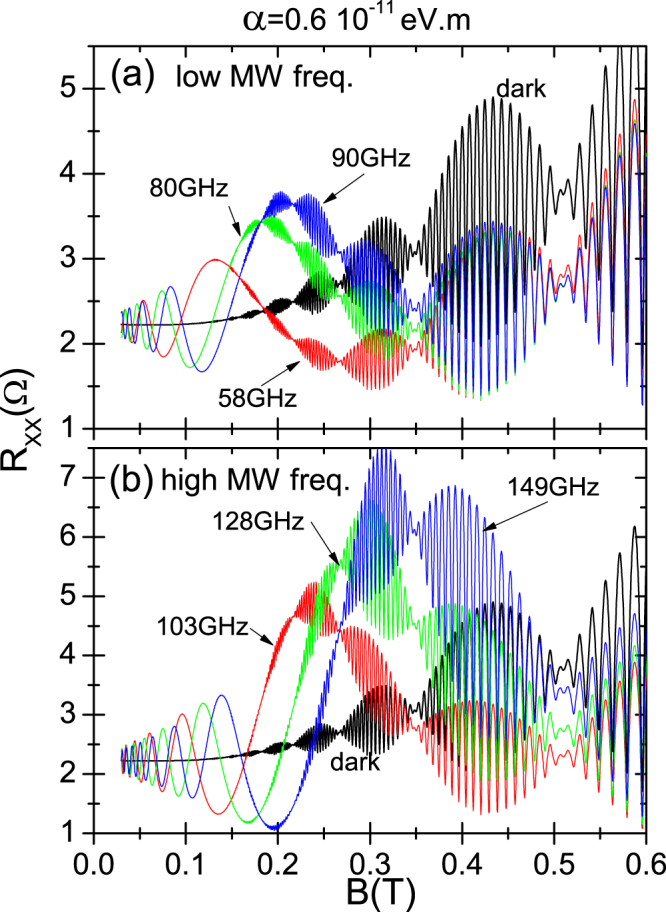
Dependence on radiation frequency, *f* of irradiated *R*_*xx*_ vs *B* for 2DES with Rashba coupling. In panel (a) we exhibit the low frequency scenario and in panel (b) the high frequency, obtaining similar results for both. Nodes *B*-position is immune to frequency and antinodes shape depends on frequency because the former depends on RIRO’s positions that, in turn, deeply depend on *f*. (T = 1 K).

In Fig. (4) we present the dependence on radiation frequency of irradiated *R*_*xx*_ for 2DES with Rashba interaction. In panel (a) we show the low frequency case and in panel (b) the high frequency, obtaining similar results for both. Thus, the nodes *B*-position turns out to be immune to radiation frequency keeping the same ones as in the dark situation. The deformation of the antinodes changes with the frequency. The reason is that the deformation or modulation depends on the RIRO position and the latter does change with radiation frequency. As a result, the same initial antinode in the dark will deform differently according to *f*. We also observe that the strongest deformation corresponds to the RIRO’s peaks irrespective of radiation frequency. The immunity of nodes with *f* can be readily explained as in the previous figure, according to Eq. (31). In this equation the nodes position depends only on the cosine terms where the Rashba term *α* shows up in the corresponding phases. In these phases *f* does not show up and then its variation will not affect the positions of either the nodes or the antinodes.

**Figure 5:**
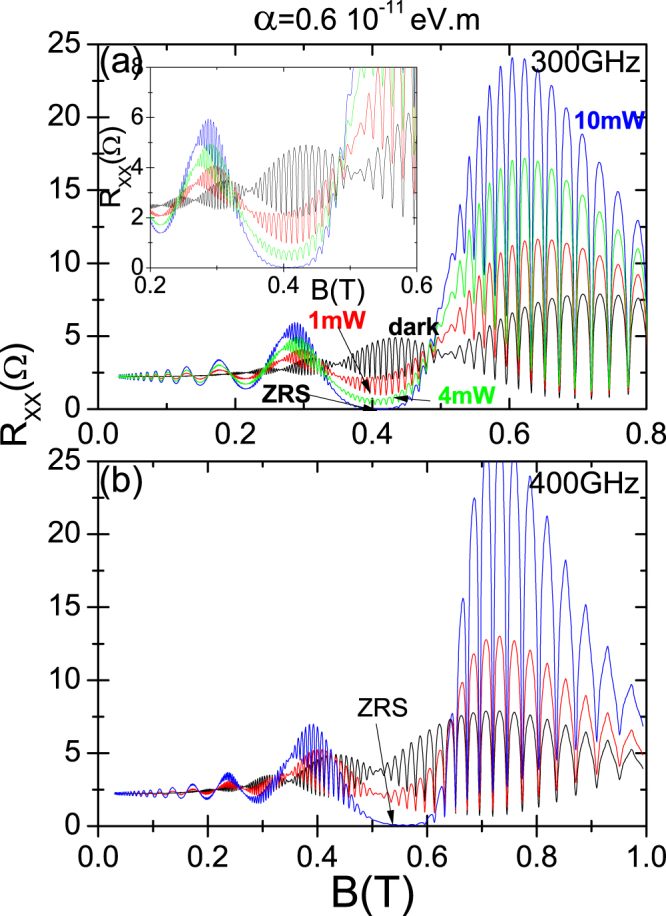
Terahertz irradiated *R*_*xx*_ versus *B* for a 2DES with Rashba interaction for radiation frequencies, *f* = 300 GHz in panel (a) and *f* = 400 GHz in panel (b). The dark case is also presented for both panels. We obtain a ZRS region in each panel as indicated by arrows. The exhibited curves in panel (a) correspond to dark and radiation power of 1 mW, 4 mW and 10 mW. We observe the evolution of the antinode of the beating pattern from dark to the onset of zero resistance states. The intensity of the *R*_*xx*_ oscillations in the antinode is diminished as the power increases. Finally these oscillations are completely wiped out at high enough power immersed in the ZRS region. The inset in this panel exhibits a zoom-in of the intermediate region of *B*: (0.2–0.6) T. In panel (b) the exhibited curves correspond to dark and radiation power of 1 mW and 10 mW. We observe the evolution of a node of the beating pattern for increasing radiation power from dark to zero resistance states. (T = 1 K).

In Fig. (5) we present the obtained results for irradiated *R*_*xx*_ vs *B* for 2DES with Rashba coupling in the terahertz regime showing two frequencies: 300 GHz in panel (a) and 400 GHz in panel (b). Apart form RIRO, due to radiation and the beating patter, we have obtained zero resistance states for $B\simeq 0.4\,{\rm{T}}$ in the upper panel and for $B\simeq 0.55\,{\rm{T}}$ in the lower panel. The ZRS region in each panel is indicated by arrows. The exhibited curves in panel (a) correspond to dark and radiation power of 1 mW, 4 mW and 10 mW. We observe the evolution of the antinode of the beating pattern from dark to the onset of zero resistance states. The intensity of the *R*_*xx*_ oscillations in the antinode is diminished as the power increases. Finally these oscillations are completely wiped out at high enough power immersed in the ZRS region. The inset in this panel exhibits a zoom-in of the intermediate region of *B*, (0.2–0.6) T, in order to observe more clearly the quenching of the antinode when approaching ZRS. Thus, we can conclude that the Rashba beatings are not simply chopped off but they are slowly quenched as the radiation power is getting bigger until they disappear and the ZRS region rise up. This behavior can be readily explained as in the previous figures, according to Eq. (31). In this equation the term of the squared brackets (radiation dependent) describes the average distance advanced by the scattered electron. As *P* increases, in the corresponding *B* region, this term is getting smaller and smaller affecting in turn the curly brackets term. The latter defines the Rashba beating that is not simply cut when approaching ZRS but modulated (diminished) by the previous radiation dependent term. In this way it is interesting to stand out that, on the other hand, when the *B* region corresponds to a RIRO peak, the intensity of the Rashba antinode is greatly enhanced by the action of radiation when the power is increased. In the lower panel we present a similar situation as in the upper panel but this time ZRS is obtained from a node. In this panel the exhibited curves correspond to dark and radiation power of 1 mW and 10 mW. Similarly as before, the node disappears as power increases, immersed in the ZRS region.
